# Co^2+^-dependent gene expression in *Streptococcus pneumoniae*: opposite effect of Mn^2+^ and Co^2+^ on the expression of the virulence genes *psaBCA*, *pcpA*, and *prtA*

**DOI:** 10.3389/fmicb.2015.00748

**Published:** 2015-07-24

**Authors:** Irfan Manzoor, Sulman Shafeeq, Tomas G. Kloosterman, Oscar P. Kuipers

**Affiliations:** ^1^Department of Molecular Genetics, Groningen Biomolecular Sciences and Biotechnology Institute, University of GroningenGroningen, Netherlands; ^2^Department of Bioinformatics and Biotechnology, Government College UniversityFaisalabad, Pakistan; ^3^Department of Microbiology, Tumor and Cell Biology, Karolinska Institutet, StockholmSweden

**Keywords:** Co^2+^, Mn^2+^, PsaR, *Streptococcus pneumoniae*, transcriptional regulation

## Abstract

Manganese (Mn^2+^)-, zinc (Zn^2+^)- and copper (Cu^2+^) play significant roles in transcriptional gene regulation, physiology, and virulence of *Streptococcus pneumoniae*. So far, the effect of the important transition metal ion cobalt (Co^2+^) on gene expression of *S. pneumoniae* has not yet been explored. Here, we study the impact of Co^2+^ stress on the transcriptome of *S. pneumoniae* strain D39. BLAST searches revealed that the genome of *S. pneumoniae* encodes a putative Co^2+^-transport operon (*cbi* operon), the expression of which we show here to be induced by a high Co^2+^ concentration. Furthermore, we found that Co^2+^, as has been shown previously for Zn^2+^, can cause derepression of the genes of the PsaR virulence regulon, encoding the Mn^2+^-uptake system PsaBCA, the choline binding protein PcpA and the cell-wall associated serine protease PrtA. Interestingly, although Mn^2+^ represses expression of the PsaR regulon and Co^2+^ leads to derepression, both metal ions stimulate interaction of PsaR with its target promoters. These data will be discussed in the light of previous studies on similar metal-responsive transcriptional regulators.

## Introduction

The Gram-positive bacterium *Streptococcus pneumoniae* resides asymptomatically in the human nasopharynx (Bogaert et al., 2004). Nonetheless, when the immune system is compromised, it can spread to different niches inside the human body (Mitchell, 2003), where it may cause serious infections like pneumonia, sepsis, otitis media, or meningitis (Obaro and Adegbola, 2002; Kadioglu et al., 2008). During its route from the nasopharynx to other parts of the human body, it is exposed to different levels of macro- and micronutrients, including varying concentrations of transition metal ions, which can affect the expression of various genes involved in virulence as well as metabolic processes (Gupta et al., 2009; Shafeeq et al., 2011a, 2013).

The transition metal ions such as manganese (Mn^2+^), zinc (Zn^2+^), copper (Cu^2+^), cobalt (Co^2+^), iron (Fe^2+^), and nickel (Ni^2+^) are indispensable components of many biological processes, forming a structural component of biomolecules, being involved in cellular and subcellular functions, and acting as catalytic cofactors in reversible oxidation–reduction and hydrolytic reactions for all forms of life (Lippard and Berg, 1994; Blencowe and Morby, 2003). An excess of metal ions can be very toxic to bacteria (Nies, 2003; Moore and Helmann, 2005). Therefore, proper homeostasis of metal ions is important for the survival of bacteria and is maintained by various transport- and eﬄux systems that are tightly regulated by metal-dependent transcriptional regulators, thus ensuring the balance of metal ions within the cell (Tottey et al., 2008; Waldron and Robinson, 2009; Lisher et al., 2013). In addition to this, the interplay of and competition between different metal ions also play an important role in the regulation of metal ion homeostasis and physiology in bacteria (Dudev and Lim, 2008; Jacobsen et al., 2011).

Co^2+^ is an important transition metal ion for many bacteria (Nies, 1992) and takes the fourth position in the Irving–Williams stability series, where the order is Mn^2+^< Fe^2+^< Co^2+^< Ni^2+^< Cu^2+^> Zn^2+^ (Irving and Williams, 1953). The concentration of Co^2+^ in the human blood and serum is 0.12 ng ml^-1^ and 0.19 ng ml^-1^, respectively (Alimonti et al., 2005). However, during certain conditions the Co^2+^ level in the human body fluids can be increased over 20 times, such as after metal-on-metal (MoM) hip arthroplasties, or upon high level exposure in regions with elevated environmental Co^2+^ levels (Jantzen et al., 2013; Cheyns et al., 2014). In pathogenic bacteria like *Mycobacterium tuberculosis* and *Staphylococcus aureus* Co^2+^-transport was linked to virulence (Remy et al., 2013; Raimunda et al., 2014). The role of Co^2+^ for *S. pneumoniae* has not been studied yet, but since there is a high number of proteins with Co^2+^-binding capacity in *S. pneumoniae*, this metal ion could be relevant for the lifestyle of this pathogenic bacterium as well (Honsa et al., 2013; Sun et al., 2013).

The transport of Co^2+^ has not been studied extensively in bacteria. The Ni^2+^/Co^2+^ permease family, NiCoT, is widely distributed in bacteria, fungi, and archaea and can transport Ni^2+^ and Co^2+^ selectively (Degen et al., 1999; Degen and Eitinger, 2002). Proteins from the NiCoT family are present in many Gram-positive bacteria including *Lactococcus lactis* and *S. thermophilus* (Lorca et al., 2007). In *Salmonella typhimurium*, the three genes *cbiN*, *cbiQ*, and *cbiO* are likely to encode an active Co^2+^-transport system (Roth et al., 1993). A comparative and functional genome analysis of the “*cbi*” system (*cbiMNQO*) showed that it constitutes a widespread Co^2+^-transport system in prokaryotes (Rodionov et al., 2006). *S. pneumoniae* also encodes the putative Co^2+^-transport genes *cbiO-I*, *cbiO-II*, and *cbiQ*, which show sequence similarity with the Co^2+^-transport genes in other prokaryotes. The Co^2+^-mediated whole genome response of *S. pneumoniae* has not been studied before. So far, the only gene of which expression was shown to be regulated by Co^2+^ is *czcD*, which is mediated by the TetR family transcriptional regulator SczA. Although mainly involved in Zn^2+^ homeostasis, CzcD slightly contributes to resistance to Co^2+^ stress as well (Kloosterman et al., 2007).

We aimed at identifying genes of which expression was influenced by a high level of Co^2+^ in *S. pneumoniae*. For this purpose, we have used whole transcriptome analysis and found a number of genes/operons that were differentially expressed under Co^2+^ stress, including *czcD*, *psaBCA*, *pcpA*, *prtA*, and *cbi*, encoding a Zn^2+^-resistance system, a Mn^2+^-uptake system, a choline binding protein, a cell-wall associated serine protease and putative Co^2+^-transport proteins, respectively. On the basis of our transcriptome analyses, β-galactosidase assays and electrophoretic mobility shift assays (EMSAs), we demonstrate that Mn^2+^ and Co^2+^ have an opposite effect on the regulation of the PsaR regulon, where Mn^2+^ represses while Co^2+^ derepressess expression of the PsaR regulon. Moreover, we show that expression of the *cbi* operon and the Zn^2+^-eﬄux gene *czcD* increases with increasing concentration of Co^2+^, suggesting a link of these genes with Co^2+^ homeostasis as well.

## Materials and Methods

### Bacterial Strains, Growth Conditions, and DNA Manipulation

Bacterial strains and plasmids used in this study are listed in **Table 1**. *S. pneumoniae* D39 wild-type (WT) was grown at 37°C in chemically defined medium (CDM) and 1% Chelex 100 resin (Bio-Rad) treated CDM (Kloosterman et al., 2006b). For 1% Chelex 100 resin (Bio-Rad) treatment, CDM was prepared without the addition of a metal mixture (a component of CDM medium) and after Chelex treatment, the metal mixture was added in the medium. The metal mixture was prepared by keeping MgCl_2_, CaCl_2_, and CuSO_4_ constant as specified in normal CDM and salts of other metal ions MnSO_4_, ZnSO_4_, CoCl_2_, FeCl_2_ and NiSO_4_ were added separately as specified in the Results section. *Escherichia coli* strain EC1000 was cultured at 37°C. Where necessary for selection, media were supplemented with the following concentrations of antibiotics: erythromycin: 0.25 μg ml^-1^ and tetracycline: 2.5 μg ml^-1^ for *S. pneumoniae*; chloramphenicol: 4 μg ml^-1^ for *L. lactis*; ampicillin: 100 μg ml^-1^ for *E. coli*. All bacterial strains used in this study were stored in 10% (v/v) glycerol at -80°C. Primers used in this study are listed in **Table 2**, and are based on the sequence of *S. pneumoniae* D39 genome. Chromosomal DNA of the *S. pneumoniae* D39 strain was used as a template for PCR amplification (Avery et al., 1995; Lanie et al., 2007).

**Table 1:** List of strains and plasmids used in this study.

Strain/plasmid	Description	Source
*** Streptococcus pneumoniae***		
D39	Serotype 2 strain, *cps 2*	Laboratory of P. Hermans
RW100	D39 Δ*psaR*	Kloosterman et al. (2008)
MP100	D39 Δ*sczA*	Kloosterman et al. (2007)
MP102	D39 Δ*czcD*	Kloosterman et al. (2007)
RW104	D39*nisRK*Δ*bgaA*::P*prtA-lacZ*; Erm^R^	Kloosterman et al. (2008)
RW109	D39*nisRK* Δ*psaR* Δ*bgaA*::P*prtA-lacZ*; Erm^R^	Kloosterman et al. (2008)
IM401	D39 Δ*bgaA*::P*nrD-lacZ*; Tet^R^	This study
IM402	D39 Δ*bgaA*::P*psaB-lacZ*; Tet^R^	This study
IM403	D39 Δ*bgaA*::P*pcpA-lacZ*; Tet^R^	This study
IM404	D39 Δ*bgaA*::P*czcD-lacZ*; Tet^R^	This study
IM405	D39 Δ*bgaA*::P*cbi-lacZ*; Tet^R^	This study
IM451	RW100 Δ*bgaA*::P*psaB-lacZ*; Tet^R^	This study
IM452	RW100 Δ*bgaA*::P*pcpA-lacZ*; Tet^R^	This study
IM453	MP100 Δ*bgaA*::P*czcD-lacZ*; Tet^R^	This study
*** Escherichia coli***		
EC1000	Km^R^; MC1000 derivative carrying a single copy of the pWV1 *repA* gene in *glgB*	Laboratory collection
*** Lactococcus lactis***		
* NZ9000*	MG1363 Δ*pepN*::*nisRK*	Kuipers et al. (1998)
*** Plasmids***		
pPP2	Amp^R^ Tet^R^; promoterless *lacZ*. For replacement of *bgaA* with promoter *lacZ* -fusion. Derivative of pTP1	Halfmann et al. (2007)
pIM301	pPP2 P*nrD-lacZ*	This study
pIM302	pPP2 P*psaB-lacZ*	This study
pIM303	pPP2 P*pcpA-lacZ*	This study
pIM304	pPP2 P*czcD-lacZ*	This study
pIM305	pPP2 P*cbi-lacZ*	This study
pRW25	pNG8048E carrying *psaR*-strep downstream of P*nisA*	Kloosterman et al. (2008)

**Table 2:** List of primers used in this study.

Name	Nucleotide Sequence (5′→3′)	Restriction site
PnrD-F	CGGAATTCCCAACAAGTAAAGACTGATTAC	EcoRI
PnrD-R	CGGGATCCGAGCCTTGTCAATCTTGTCC	BamHI
PprtA-F	CATGGAATTCATCTCTTCAAACCACGTCAACGTCGC	EcoRI
PprtA-R	CATGGGATCCTTATCTACTACTACTTTTTCTTTATCA	BamHI
PpsaB-F	CGGAATTCTTCCAAGTTTTTTACACTTG	EcoRI
PpsaB-R	CGGGATCCATTGTTGGTCCATGGAGCAC	BamHI
PpcpA-F	CGGAATTCCCTTCAAATTTTAAGTCC	EcoRI
PpcpA-R	CGGGATCCGTTAATGATAATATTGTAG	BamHI
PczcD-F	CGGAATTCTAGATGGCTTTTTTGGTTTTGCTG	EcoRI
PczcD-R	CGGGATCCGCAGACTCAGAATAGACTCATTC	BamHI
PadcR-F	CGGAATTCTTTTTCAGCAAAGATTGGG	EcoRI
PadcR-R	CGGGATCCCTTTCCTTTTAGACTTCTC	BamHI
PnrdH-F	GCATGAATTCCCACTACGTGGAAATCTTTAG	EcoRI
PnrdH-R	CATGGGATCCGCTTGGTCATTTTACATTGGAC	BamHI
Pcbi-F	CATGGAATTCCCTCAATCTTTGGTATTATACC	EcoRI
Pcbi-R	CATGGGATCCCCATGCACTAACTCCATG	BamHI

### Reverse Transcription (RT)-PCR

**FIGURE 1:**
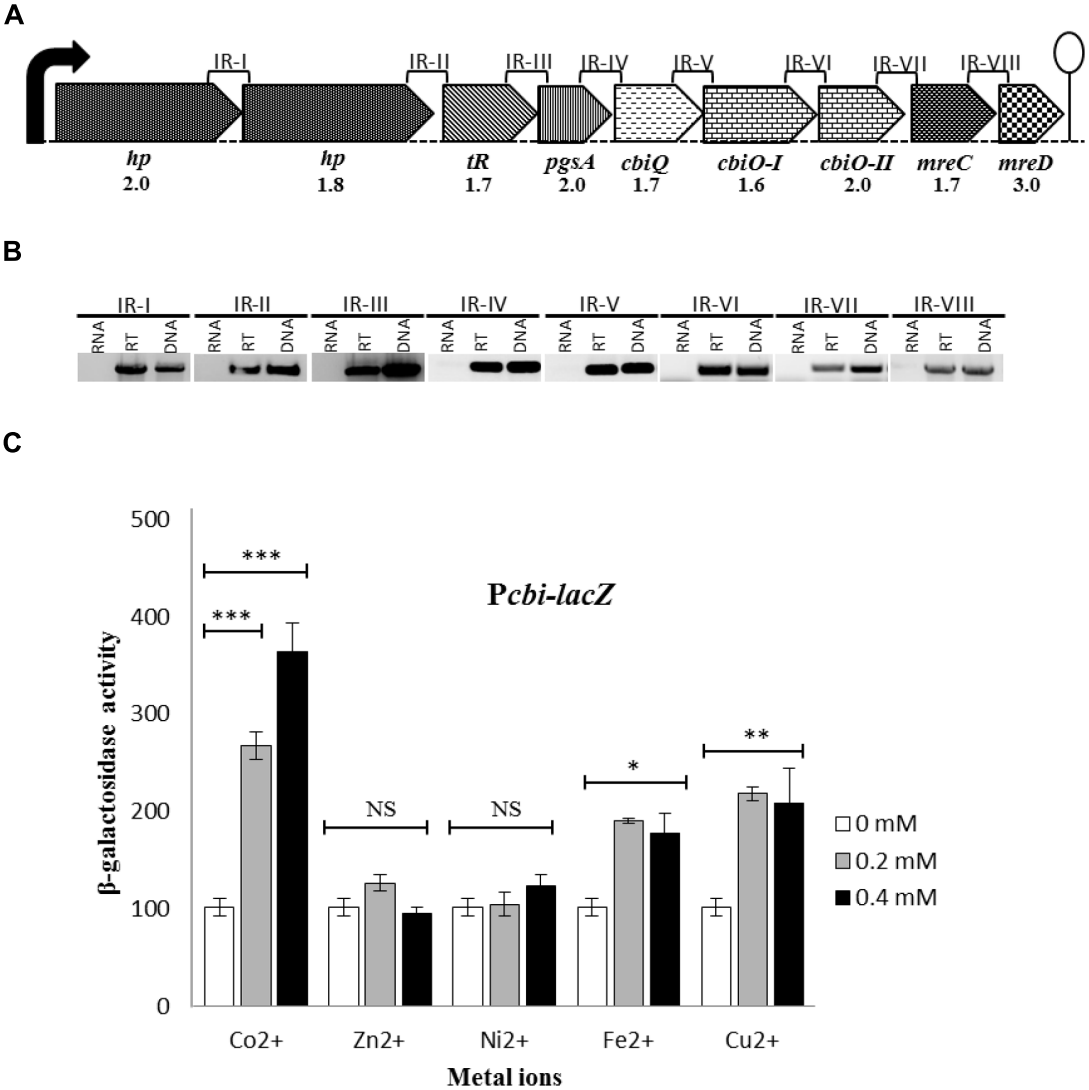
**(A)** Organization of the putative *cbi* operon. The lollipop indicates the putative transcriptional terminator. Black arrow represents the promoter region. Expression ratios of the *cbi* operon as determined by the transcriptome analysis [chemically defined medium (CDM) + 0.5 mM Co^2+^/CDM + 0 mM Co^2+^] are indicated underneath. **(B)** Confirmation of the polycistronic nature of the *cbi* operon by Reverse Transcription (RT)-PCR analysis. Total RNA was isolated from the D39 wild-type (WT) grown in CDM + 0.5 mM Co^2+^. RT-PCR was performed with and without RT treatment using primer pairs for the intergenic regions IR-I to IR-VIII. Genomic DNA of D39 WT was taken as a positive control. **(C)** Expression level (in Miller units) of a P*cbi-lacZ*-fusion in D39 WT in CDM supplemented with different concentrations of Co^2+^, Ni^2+^, Zn^2+^, Fe^2+^, and Cu^2+^. SD of three independent replications is indicated with error bars. Statistical significance of the differences in the expression levels was determined by one-way ANOVA (NS, not significant, ^∗^*P* < 0.05, ^∗∗^*P* < 0.001, and ^∗∗∗^*P* < 0.0001).

To confirm that the *cbi* gene cluster transcribes as a single transcriptional unit, D39 WT was grown in CDM with 0.5 mM Co^2+^ and total RNA was isolated from cells grown till mid-exponential phase of growth (OD_600_ = 0.2) as described (Shafeeq et al., 2015). In short, cells were harvested by centrifugation for 2 min at 10,000 rpm at 4°C. Cell pellets were resuspended in 400 μl of nuclease free water (DEPC-treated), after which 50 μl of 10% SDS, 500 μl of phenol/chloroform (1:1) and 500 mg glass beads were added. Total RNA was isolated using the Roche RNA isolation Kit. DNA contamination was eliminated from the RNA sample by treatment with 2 U of RNase free DNase I (Invitrogen, Paisley, UK). cDNA samples were prepared by using superscript III RT and random nanomers at 42°C for 16 h. The intergenic regions IRI to IRVII (**Figure 1**) were amplified by primer pairs mentioned in **Table 2**. For fair comparison of PCR products, 100 ng of RNA and 30 ng of DNA were used.

### Inductively Coupled Plasma-Mass Spectrometry (ICP-MS) Analysis

For Inductively coupled plasma-mass spectrometry (ICP-MS) analysis, *S. pneumoniae* strain D39 was grown in CDM with and without 0.5 mM Co^2+^ till mid-exponential phase (OD_600_ = 0.25 for 0 mM Co^2+^ and OD_600_ = 0.2 for 0.5 mM Co^2+^) of growth. Cultures were centrifuged and washed (at 4°C) twice with the CDMchelex medium and twice with overnight Chelex (Sigma) treated phosphate-buffered saline (PBS) with 1 mM nitrilotriacetic. The cell pellets were dried overnight in a Speedvac at room temperature. The dried cells were lysed in 2.5% nitric acid (Ultrapure, Sigma–Aldrich) for 10 min at 95°C by vigorous vortexing after 15 s. The lysed cell samples were used for ICP-MS analysis as described before (Jacobsen et al., 2011). Moreover, ICP-MS analysis was also performed on CDM, CDMchelex, CDM-Mn^2+^, and CDMchelex-Mn^2+^ media. Metal ion concentrations were expressed as μg g^-1^ dry weight of cells and in μg l^-1^ in case of plain CDM.

### Construction of *lacZ*-Fusions and β-Galactosidase Assays

Transcriptional *lacZ*-fusions to the promoters of *nrdD*, *psaB*, *pcpA*, *czcD*, and the *cbi* operon were constructed in pPP2 (Halfmann et al., 2007) with the primer pairs listed in **Table 2**. The PCR products were digested and cloned into the *EcoRI*/*BamHI* sites of pPP2 resulting in plasmids pIM301-305. *E. coli* EC1000 was used as cloning host. The pIM301-305 were transformed into D39 WT resulting in strains IM401-406, whereas pIM302-303 were also introduced into the RW100 (Δ*psaR*) strain (Kloosterman et al., 2008), resulting into strains IM451-452. pIM305 was introduced into MP100 (Δ*sczA*) strain (Kloosterman et al., 2007), resulting into strain IM453. All plasmids were checked for the presence of the correct insert by means of PCR and DNA sequencing.

For β-galactosidase assays, derivatives of *S. pneumoniae* D39 were grown in triplicate in CDM and CDMchelex at 37°C supplemented with different metal ion concentrations as mentioned in the “Results” section. Cells were harvested at the mid-exponential phase of growth (OD_600_ = 0.2). Cells were treated with mixture of Z-buffer [Na_2_HPO_4_^∗^2H_2_O (60 mM), NaH_2_PO_4_^∗^H_2_O (40 mM), KCl (10 mM), MgSO_4_^∗^7H_2_O (1 mM)] and CTAB (Cetyltrimethylammonium bromide). Four milligram per milliliter of ortho-Nitrophenyl-β-galactoside (ONPG) was added to start the reaction. The reactions were stopped with the addition of Na_2_CO_3_ and β-galactosidase activity was measured as described before (Kloosterman et al., 2006b). SD for each sample was calculated from three independent biological replicates.

### Overexpression and Purification of Strep-Tagged PsaR

The overexpression of C-terminally Strep-tagged PsaR (Kloosterman et al., 2008) was achieved by mean of the nisin-inducible system (NICE) in *L. lactis* strain NZ9000 (Kuipers et al., 1998). Cells were grown till an OD_600_ of 0.5 and then induced with 5 ng ml^-1^ nisin in 1 L culture. After 2 h of nisin induction, the cell culture was harvested and resuspended in 10 ml buffer A (0.25 M NaCl, 10 mM MgCl_2_, 2 0 mM Tris-HCl, pH 8, 10% glycerol, 1 mM β-mercaptoethanol) with 1 mg/ml lysozyme and one tablet of protease inhibitor cocktail (Complete Mini, Roche). After half hour incubation at 30°C cells were sonicated on ice and cell debris was removed by centrifugation. Purification of PsaR-Strep was performed using the Streptactin column from IBA according to the supplier’s instructions (www.iba-go.com). Buffers without EDTA were used, and the purified protein was stored at a concentration of 0.2 mg/ml in the elution buffer (100 mM Tris-HCl [pH 8], 150 mM NaCl, 2.5 mM desthiobiotin, 1 mM β-mercaptoethanol) with 10% glycerol at -80°C.

### Electrophoretic Mobility Shift Assays

Electrophoretic mobility shift assays were performed as described (Kloosterman et al., 2008). In short, PCR products of P*psaB*, P*pcpA*, P*prtA*, and P*adcR* were labeled with [γ-^33^P] ATP. EMSAs were carried out in buffer containing 20 mM Tris-HCl (pH 8.0), 5 mM MgCl_2_, 8.7% (w/v) glycerol, 62.5 mM KCl. 25 μg/ml bovine serum albumin, 25 μg/ml poly(dI-dC) and 5000 cpm of [γ-^33^P] ATP-labeled PCR product. Reactions were incubated at 30°C for 30 min before loading on gels. Gels were run in 1 M Tris-borate buffer (pH 8.3) at 95 V for 90 min.

### DNA Microarray and Data Analysis

For DNA microarray analysis to investigate Co^2+^ stress, the transcriptome of *S. pneumoniae* D39 WT, grown in 2 biological replicates in CDM with 0.5 mM CoCl_2_ was compared to the transcriptome of the same strain grown in two biological replicates in CDM with 0 mM CoCl_2_. Cells were harvested at mid-exponential phase of growth by means of centrifugation for 2 min at 11000 rpm at 4°C and immediately frozen in liquid nitrogen. RNA isolation and cDNA synthesis was performed as described before (Shafeeq et al., 2015). Cy3/Cy5 labeling of cDNA was performed with the Cyscribe post Labeling Kit (Amersham Bioscience). Hybridization was performed with labeled cDNA for 16 h at 45°C in Ambion SlideHyb #1 hybridization buffer on super amine glass slides (Array-It, SMMBC). Slides were scanned with a Genepix 4200 laser scanner at 10 μm resolution. Genepix software (GenePix^®^ Pro 7) was used to analyze the slides. The *MicroPrep* software package was used to analyze the raw data further. The expression ratio of the signals of D39 grown in CDM with 0.5 mM Co^2+^ over D39 grown in CDM with 0 mM Co^2+^ was calculated from the data of at least seven spots by Cyber-T. A gene was considered differentially expressed when the Bayesian *p*-value <0.001 and a fold change cut-off of 2 was applied. The transcriptomic data have been submitted to GEO (Gene Expression Omnibus) with accession number GSE57696.

## Results

### Organization of the *cbi* Operon in *S. pneumoniae* D39

The important transition metal ion Co^2+^ is usually taken up by bacterial cells from the outer environment through specific transport systems like CbiMNQO (Roth et al., 1993; Rodionov et al., 2006). BLAST searches with the CbiMNQO sequences revealed the presence of putative Co^2+^-transport genes in *S. pneumoniae* D39 as well (Supplementary Figure S1). These genes are organized within a cluster of nine genes (*SPD2052-2044*) and most likely this cluster of genes is transcribed as a single transcriptional unit (**Figure 1A**). Due to high homology of these genes with putative Co^2+^-uptake genes in other bacteria, here, we will name this operon as a *cbi* operon.

The first two genes (*SPD2052–SPD2051*) of the *cbi* operon encodes a peptidase, i.e., a M16 family protein, and a hypothetical protein, which have homology with Zn^2+^-dependent proteases and peptidases, respectively. The third gene (*SPD2050*) of the *cbi* operon is a putative transcriptional regulator that belongs to the XRE (Xenobiotic Response Element) family of transcriptional regulators. This is the second most occurring family of transcription regulators in bacteria and has diverse metabolic functions. The fourth gene in the *cbi* operon is *pgsA* (*SPD2049*) encoding CDP-diacylglycerol-glycerol-3-phosphate 3-phosphatidyltransferase. This gene is involved in phospholipid biosynthesis in *Rhodobacter sphaeroides* and is predicted to be essential for the survival of *Streptococcus mutans* (Dryden and Dowhan, 1996; MacGilvray et al., 2012). Downstream of *pgsA*, three genes, i.e., *cbiO-I*, *cbiO-II*, and *cbiQ* (*SPD2048*–*SPD2046*) are present. *cbiO-I* and *cbiO-II* putatively encode a Co^2+^ ATP-binding transporter subunit, whereas *cbiQ* encodes a Co^2+^-ABC transporter permease. BLAST searches showed that the *cbiO-I* and *cbiO-II* genes of *S. pneumoniae* are conserved in different streptococci and also have a sequence similarity with the *cbiO* gene of other bacteria, a gene that has been shown to be involved in Co^2+^-transport (Roth et al., 1993; Rodionov et al., 2006). Following *cbiQ*, *mreC*, and *mreD* (*SPD2045–SPD2044*) that encode rod shape-determining proteins, are present. MreC and MreD are involved in the synthesis of peptidoglycan in many bacteria (Carballido-López and Formstone, 2007; Bendezu and de Boer, 2008). In *Bacillus subtilis*, the overexpression of *mreC* and *mreD* stimulate protease production (Kubo et al., 1996). In *S. pneumoniae*, the exact role of MreC and MreD is not known yet, but both of the genes have been shown to be essential for the growth of *S. pneumoniae* D39 (Land and Winkler, 2011).

To confirm that the *cbi* operon is transcribed as a single transcriptional unit, all the intergenic regions between these nine genes (IRI to IRVIII) were analyzed by RT-PCR (**Figure 1B**). Our RT-PCR data showed that the *cbi* operon is indeed transcribed as a single transcriptional unit (**Figure 1B**). Moreover, recent RNA-seq data of *S. pneumoniae* also support our RT-PCR data that the *cbi* operon is transcribed as a single transcriptional unit starting from *SPD2052* and ending with *SPD2044* (GSE54199; Slager et al., 2014).

### Co^2+^-Dependent Expression of the *cbi* Operon in *S. pneumoniae* D39

The presence of three putative Co^2+^-transport genes in the *cbi* operon suggests its putative role in metal ion transport. To investigate the effect of different concentrations of various metal ions (Co^2+^, Ni^2+^, Zn^+2^, Fe^2+^, and Cu^+2^) on the expression of the *cbi* operon (as measured by an ectopic P*cbi-lacZ* transcriptional fusion) of *S. pneumoniae*, we performed β-galactosidase assays. The measurement of β-galactosidase activity revealed that expression of P*cbi-lacZ* increased significantly in the presence of Co^2+^ compared to other tested metal ions. Fe^2+^ and Cu^2+^ also slightly increase the expression of P*cbi-lacZ* compared to the control (**Figure 1C**), but the increase in the expression of P*cbi-lacZ* to Fe^2+^ and Cu^2+^ was less compared to Co^2+^. These data suggest that the expression of the *cbi* operon is Co^2+^-dependent.

### Identification of Co^2+^-Dependent Genes in *S. pneumoniae*

To investigate the genome-wide influence of Co^2+^ stress in *S. pneumoniae*, a transcriptome analysis was performed using the D39 WT strain grown in CDM (Kloosterman et al., 2006a) either with 0.5 mM or 0 mM Co^2+^ (**Table 3**). Expression of the *cbi* operon encoding (putative) Co^2+^ transporters was affected by Co^2+^ (**Figure 1A**). Upregulation of the *cbi* operon further confirmed the Co^2+^-dependent expression of the *cbi* operon as seen with the β-galactosidase assays mentioned above. Identification of the gene encoding the Zn^2+^-eﬄux system CzcD in the Co^2+^ responsive set of genes, as well as the downstream gene *adhC*, encoding a Zn^2+^-containing alcohol dehydrogenase, is in agreement with previous findings (Kloosterman et al., 2007). The same holds for upregulation of the *nrd* operon, involved in synthesis of deoxyribonucleoside triphosphate (Jordan and Reichard, 1998; Torrents et al., 2001), by Co^2+^ stress (Kloosterman et al., 2007).

**Table 3:** Summary of transcriptome comparison of *S. pneumoniae* D39 wild-type grown in chemically defined medium (CDM) plus 0.5 mM Co^2+^ and CDM plus 0 mM Co^2+^.

Gene tag^a^	Function^b^	Ratio^c^	*P*-value
*SPD0053*	Amidophosphoribosyltransferase	2.0	2.09E-06
*SPD0054*	Phosphoribosylformylglycinamidine cyclo-ligase	2.0	5.29E-08
*SPD0055*	Phosphoribosylglycinamide formyltransferase	2.2	1.12E-05
*SPD0056*	VanZ protein	2.4	6.93E-05
*SPD0057*	Bifunctional purine biosynthesis protein, PurH	2.5	1.28E-07
*SPD0187*	Anaerobic ribonucleoside-triphosphate reductase, NrdD	11.2	2.93E-14
*SPD0188*	Hypothetical protein	4.3	2.47E-10
*SPD0189*	Acetyltransferase, GNAT family protein	11.0	5.44E-10
*SPD0190*	Anaerobic ribonucleoside-triphosphate reductase, NrdG	10.5	1.22E-15
*SPD0191*	Hypothetical protein	8.3	1.34E-07
*SPD0458*	Heat-inducible transcription repressor, HrcA	2.5	1.09E-10
*SPD0459*	Heat shock protein, GrpE	2.1	4.82E-09
*SPD0558*	Cell wall-associated serine protease, PrtA	16.6	3.89E-14
*SPD1461*	Mn^2+^ ABC transporter, ATP binding protein, PsaB	8.7	2.18E-14
*SPD1462*	Manganese ABC transporter, permease protein, PsaC	8.7	2.18E-14
*SPD1594*	XRE family Transcriptional regulator	3.1	7.14E-09
*SPD1636*	Zn^2+^-containing alcohol dehydrogenase	8.4	2.05E-14
*SPD1637*	MerR family transcriptional regulator	11.0	1.41E-10
*SPD1638*	Cation eﬄux system, CzcD	20.8	0
*SPD1965*	Choline binding protein, PcpA	5.0	5.48E-06
*SPD2044*	Rod shape-determining protein, MreD	3.0	8.75E-11
*SPD2046*	Co^2+^ ABC transporter, permease protein, CbiQ	2.0	1.22E-09
*SPD2049*	CDP-diacylglycerol-glycerol-3-phosphate 3-phosphatidyltransferase PgsA	2.0	1.13E-06
*SPD2052*	Hypothetical protein	2.0	6.84E-09

*^a^Gene numbers refer to D39 locus tags. ^b^D39 annotation/TIGR4 annotation. (Hoskins et al., 2001; Lanie et al., 2007), ^c^Ratios (0.5 mM Co^2+^/0 mM Co^2+^).*

Interestingly, a number of virulence genes were upregulated by Co^2+^ stress, namely *psaBCA*, *pcpA*, and *prtA*, encoding a Mn^2+^-dependent ABC transporter (McAllister et al., 2004), a choline binding protein (Sánchez-Beato et al., 1998) and a serine protease (Bethe et al., 2001; Mirza et al., 2011), respectively. The regulation of these genes has been shown before to be dependent on the balance between the concentrations of Mn^2+^ and Zn^2+^ *via* the transcriptional regulator PsaR (Johnston et al., 2006; Kloosterman et al., 2008; Honsa et al., 2013). Based on our observation, we speculated PsaR to play a role in the regulation of these genes in the presence of Co^2+^ as well.

Two transcriptional regulators showed increased expression under Co^2+^ stress. *SPD1594*, belonging to the XRE family that has homology with a Zn^2+^-dependent peptidase, was previously found to be upregulated during Zn^2+^-limitation (Shafeeq et al., 2011a), suggesting that Co^2+^ and Zn^2+^ have opposite effects on its expression. The second, HrcA, a heat-inducible transcription repressor, has previously been shown to be involved in repression of *dnaK*, a chaperone protein, and *groE*, a chaperonin, in the presence of Ca^2+^ (Kwon et al., 2005; Kim et al., 2008). Upregulation of the HrcA regulon may indicate that a high concentration of Co^2+^ causes stress to the cell. Overall, Co^2+^ stress induces broad transcriptomic changes in *S. pneumoniae*.

### Effect of Co^2+^ on the PsaR-Mediated Expression of the Virulence Genes *pcpA*, *psaBCA*, and *prtA*

**FIGURE 2:**
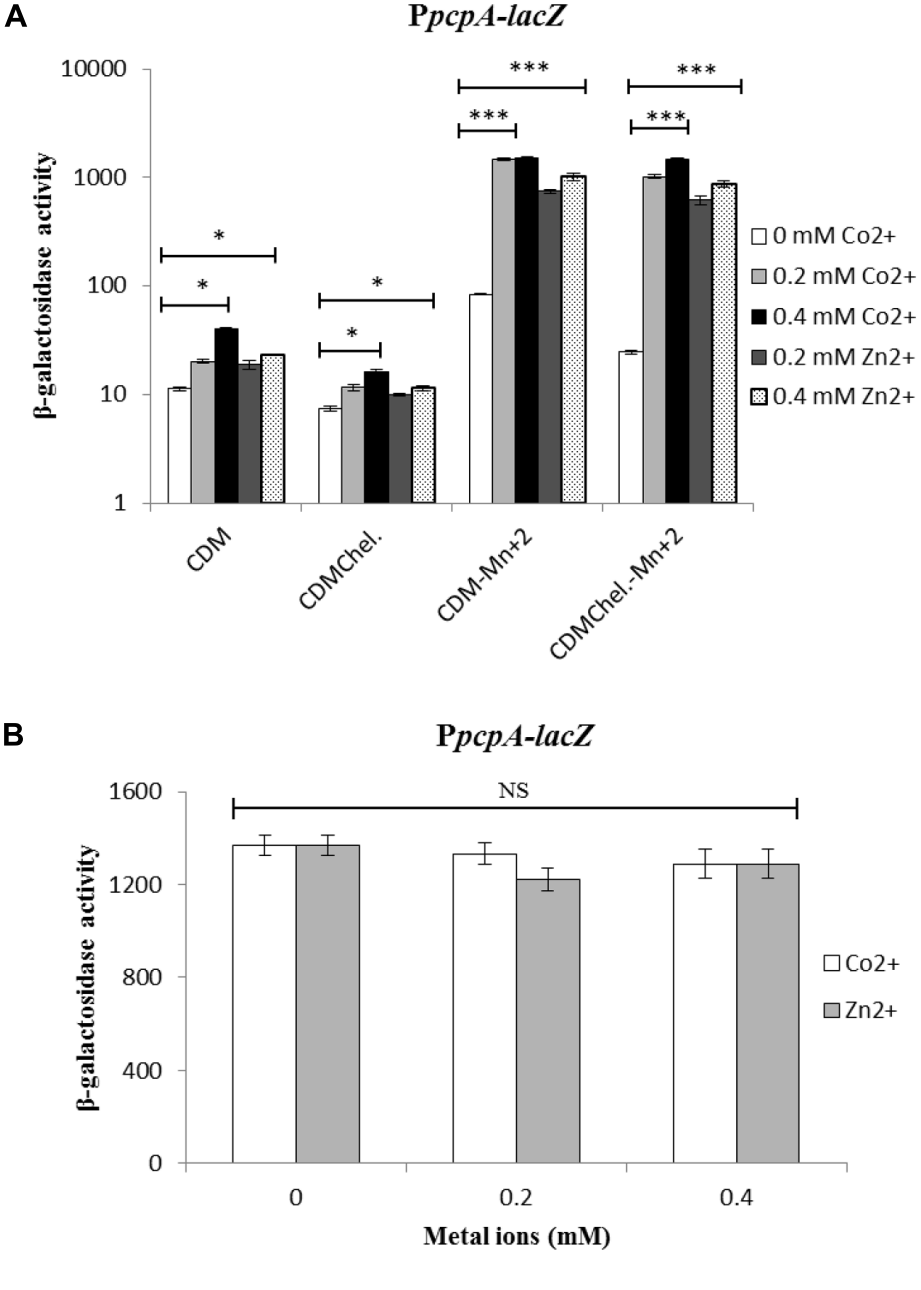
**(A)** Expression level (in Miller units) of P*pcpA-lacZ* in D39 WT in CDM, CDM-Mn^2+^, CDMchelex, and CDMchelex-Mn^2+^ supplemented with different concentrations of Co^2+^ and Zn^2+^. **(B)** Expression level (in Miller units) of P*pcpA-lacZ* in Δ*psaR* strain. SD of three independent replications is indicated with error bars. Statistical significance of the differences in the expression levels was determined by one-way ANOVA (NS, not significant, ^∗^*P* < 0.05, and ^∗∗∗^*P* < 0.0001).

To investigate the transcriptional response of *prtA*, *pcpA*, and *psaBCA* under different Co^2+^ concentrations, transcriptional *lacZ-*fusions with the respective promoters of *pcpA* and *psaBCA* were constructed in plasmid pPP2 (Halfmann et al., 2007) and *prtA* transcriptional *lacZ-*fusion was constructed in pORI13 (Kloosterman et al., 2008), and introduced into D39 WT. The strains containing the P*pcpA*-*lacZ*, P*psaB*-*lacZ*, and P*prtA*-*lacZ* transcriptional fusions were grown in CDM (Complete CDM), CDM-Mn^2+^ (CDM without Mn^2+^), CDMchelex (Complete CDM treated with Chelex) and CDMchelex-Mn^2+^ (CDM without Mn^2+^ treated with Chelex) with addition of Co^2+^ and Zn^2+^. Mn^2+^-depleted medium was used, since the binding affinity of Mn^2+^ with PsaR is very high compared to other metal ions (Zn^2+^ or Co^2+^; Lisher et al., 2013). The measurement of β-galactosidase activity revealed higher expression of the P*pcpA-lacZ*, P*psaB-lacZ*, and P*prtA-lacZ* transcriptional fusions in Mn^2+^-depleted medium (CDM-Mn^2+^ and CDMchelex-Mn^2+^) compared to CDM/CDMchelex, which might be due to Mn^2+^ starvation (**Figure 2A** and Supplementary Figure S2). The further addition of Co^2+^/Zn^2+^ leads to higher expression of P*pcpA-lacZ*, P*psaB-lacZ*, and P*prtA-lacZ* (**Figure 2A** and Supplementary Figure S2), suggesting that the derepression of the PsaR regulon is not specific to Mn^2+^ starvation but also Co^2+^ and Zn^2+^ have a derepressive effect. We also observed that at the same concentration of Co^2+^ and Zn^2+^ the effect of Co^2+^ on the expression of the PsaR regulon is stronger compared to that of Zn^2+^.

It is likely that, besides the response to Mn^2+^ and Zn^2+^ (Kloosterman et al., 2008), PsaR also mediates the expression of P*pcpA-lacZ*, P*psaB-lacZ*, and P*prtA-lacZ* in the presence of Co^2+^. To verify this hypothesis, the P*pcpA*-*lacZ*, P*psaB*-*lacZ*, and P*prtA*-*lacZ* were transformed into the clean knockout strain of *psaR* (Kloosterman et al., 2008). β-galactosidase activity showed that the expression of P*pcpA-lacZ*, P*psaB-lacZ*, and P*prtA-lacZ* were derepressed in the Δ*psaR* strain and independent of the presence of Co^2+^ (**Figure 2B** and Supplementary Figure S3).

**FIGURE 3:**
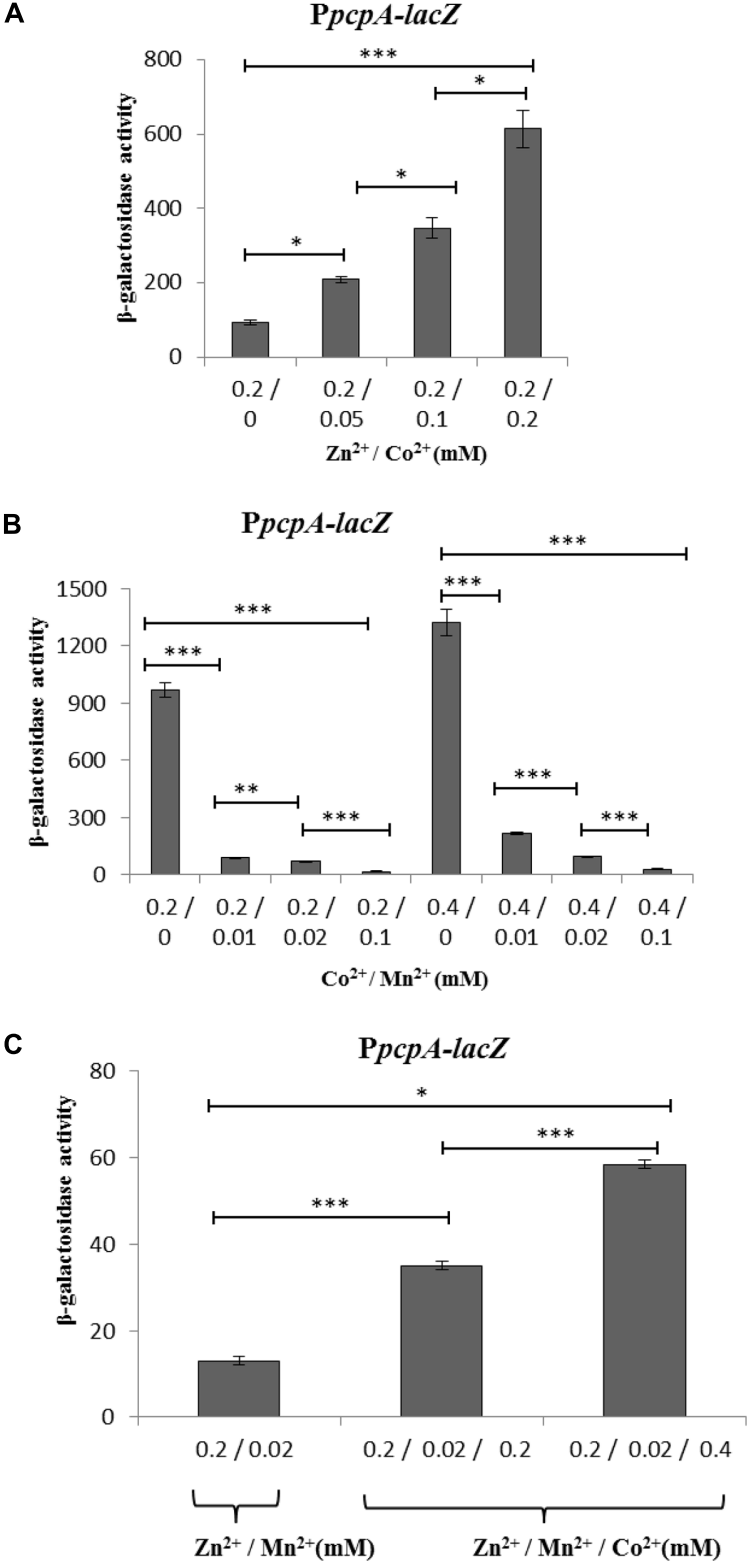
**Expression level (in Miller units) of P*pcpA-lacZ* in D39 WT in CDM (without Mn^2+^ addition) supplemented with different concentrations of Co^2+^ and Zn^2+^**(A)**, with different concentrations of Co^2+^ and Mn^2+^**(B)**, and with different concentrations of Co^2+^, Mn^2+^, and Zn^2+^**(C)**.** SD of three independent replications is indicated with error bars. Statistical significance of the differences in the expression levels was determined by one-way ANOVA (NS, not significant, ^∗^*P* < 0.05, ^∗∗^*P* < 0.001, and ^∗∗∗^*P* < 0.0001).

To determine the effect of Co^2+^ in conjunction with other metal ions on gene expression mediated by PsaR, β-galactosidase activity of P*pcpA-lacZ* and P*psaB-lacZ* was measured using different concentrations of Co^2+^, Mn^2+^, and Zn^2+^. This showed that the expression of P*pcpA-lacZ* increased with a gradual increase in the concentration of Co^2+^ at a constant concentration of Zn^2+^ (**Figure 3A**) in CDM-Mn^2+^. 0.2 mM of Zn^2+^ was used because at this concentration maximum expression of P*pcpA*-*lacZ* was observed. It has been shown previously that Mn^2+^ can inhibit the activation of the PsaR regulon by PsaR even in the presence of Zn^2+^. Therefore, we decided to also check if Co^2+^ and Mn^2+^ influence each other’s effects. We kept the concentration of Co^2+^ constant and gradually increased the concentration of Mn^2+^ from 0.01 mM to 0.1 mM. As expected, the expression of P*pcpA-lacZ* was decreased with increasing concentrations of Mn^2+^ (**Figure 3B**). This indicates that Mn^2+^ can also nullify the Co^2+^-dependent depression of the PsaR regulon. However, this Mn^2+^-dependent repression of the PsaR regulon in the presence of Co^2+^ is less strong compared to when Zn^2+^ is present instead.

In further experiments, we kept the concentration of Mn^2+^ and Zn^2+^ constant, while increasing the concentration of Co^2+^ in the medium and performed β-galactosidase assays with P*pcpA-lacZ*. β-galactosidase activity showed that the expression of P*pcpA-lacZ* was increased with increasing concentrations of Co^2+^ at a constant concentration of Mn^2+^ and Zn^2+^ (**Figure 3C**). The same trend of expression was also observed for P*psaB-lacZ* (Supplementary Figure S4). Next to using *lacZ* transcriptional reporter assays, qRT-PCR data of the genes in the PsaR regulon in the presence of high Co^2+^ are also in line with our transcriptome analysis (Supplementary Table S1).

### ICP-MS Analysis of Intracellular Mn^2+^ and Co^2+^ in *S. pneumoniae*

To quantify the exact concentration of metal ions in the media that were used in this study, ICP-MS analysis was performed. Concentrations of different metal ions measured by ICP-MS in CDM, CDMchelex, CDM-Mn^2+^, and CDMchelex-Mn^2+^ are summarized in Supplementary Table S2. ICP-MS analysis revealed the concentration of Mn^2+^ present in CDM and CDMChelex is 5–7 μM, which is already enough to cause Mn^2+^-dependent repression of *psaBCA*, *pcpA*, and *prtA* by PsaR (Kloosterman et al., 2008), and also explains why we did not observe a big difference in the expression of *psaBCA*, *pcpA*, and *prtA* between CDM and CDMchelex.

To investigate whether the transcriptome effects observed above in the presence of 0.5 mM Co^2+^ correlate with a high cell-associated concentration of Co^2+^, ICP-MS analysis was performed on whole cell extract. For this purpose, cells were grown in CDM without and with 0.5 mM added Co^2+^. The concentrations of cell-associated metal ions in *S. pneumoniae* D39 grown with or without 0.5 mM Co^2+^ are given in **Table 4**. Interestingly, no difference in the cell-associated amount of Mn^2+^ was observed in the cells grown in medium with 0 mM Co^2+^ compared to the cells grown in medium with 0.5 mM Co^2+^. Thus, the effect of Co^2+^ on the expression of the PsaR regulon might be direct, rather than indirectly *via* a change in the intracellular concentration of Mn^2+^.

**Table 4:** Intracellular metal ion concentrations (μg g^-1^) of *S. pneumoniae* D39 grown in complete CDM with 0 mM and 0.5 mM Co^2+^.

Metal ions	0 mM Co^2+^	0.5 mM Co^2+^
Mn^2+^	26	28
Zn^2+^	22	24
Co^2+^	< 1	44
Fe^2+^	<1	<1
Ni^2+^	<1	<1

*Data are the mean of two independent experiments.*

### Binding of PsaR to its Target is Mn^2+^ and Co^2+^-Dependent

**FIGURE 4:**
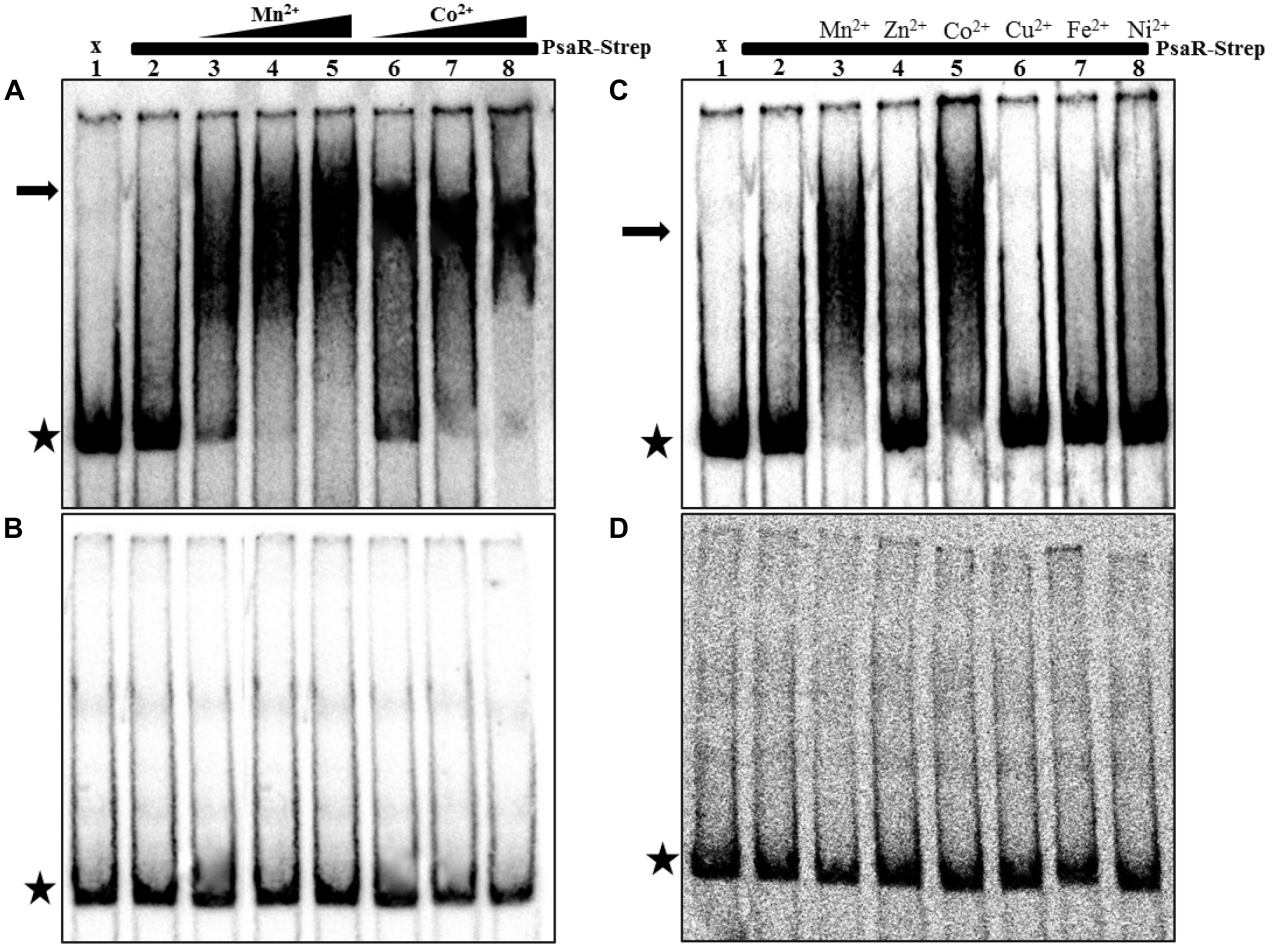
***In vitro* interaction of PsaR-Strep with the promoter region of *pcpA*.** PsaR-Strep was added at concentration of 30 nM as indicated with horizontal bar above the lanes, while lane 1 is without protein. Arrows indicate the position of the shifted probe and asterisks indicate the position of the free probe. **(A)** Mn^2+^ was added in concentrations of 0.05 mM, 0.1 mM, and 0.2 mM as indicated by the triangular bar above lanes 3, 4, and 5. Co^2+^ was added in concentrations of 0.05 mM, 0.2 mM, and 0.4 mM as indicated by the triangular bar above lanes 6, 7, and 8, respectively. **(B)** The same experiment was done as described in **(A)**, except that now 100 mM EDTA was added. **(C)** Metal ions were added as indicated above the lanes in concentrations of 0.2 mM. **(D)** The same experiment was done as described in **(A)**, except that P*adcR* was used (negative control) instead of P*pcpA*.

To find out whether Co^2+^ directly affects the interaction of PsaR binding with P*psaB*, P*pcpA*, and P*prtA*, EMSAs were performed with purified Strep-tagged PsaR (PsaR-Strep) protein and ^33^P-labeled promoter regions of *psaB*, *pcpA*, *prtA*, and *adcR*. The promoter region of *adcR* was taken as negative control. PsaR-Strep was unable to shift the promoter region of *pcpA* in the absence of metal ions (Lane 2 in **Figure 4A**). In the presence of 0.05 mM Mn^2+^ a slight shift was seen, while at 0.1 mM and 0.2 mM Mn^2+^, a complete shift of the promoter region of *pcpA* was observed (Lanes 3, 4, and 5 in **Figure 4A**), which is in agreement with a previous study (Kloosterman et al., 2008). Interestingly, PsaR-Strep also shifted the *pcpA* promoter fragment in the presence of 0.05 mM, 0.2 mM and 0.4 mM Co^2+^ (Lanes 6, 7, and 8 in **Figure 4A**). No shift was observed when the EMSAs were done in the presence of EDTA in reactions with otherwise the same conditions (**Figure 4B**). EMSAs were also performed in the presence of different metal ions, including Mn^2+^, Co^2+^, Zn^2+^, Cu^2+^, Fe^2+^, and Ni^2+^. No shift was observed with the tested metal ions except with Mn^2+^ and Co^2+^, which indicate that the PsaR-promoter interaction occurs specifically with Mn^2+^ and Co^2+^ (**Figure 4C**). Under the same conditions we did not see any shift with P*adcR* as a negative control (**Figure 4D**). EMSAs were also done with P*psaB* and P*prtA*, giving similar results as with P*pcpA* (Supplementary Figure S5).

### Effect of Co^2+^on the Expression of the *nrd* and *czcD* Operons

**FIGURE 5:**
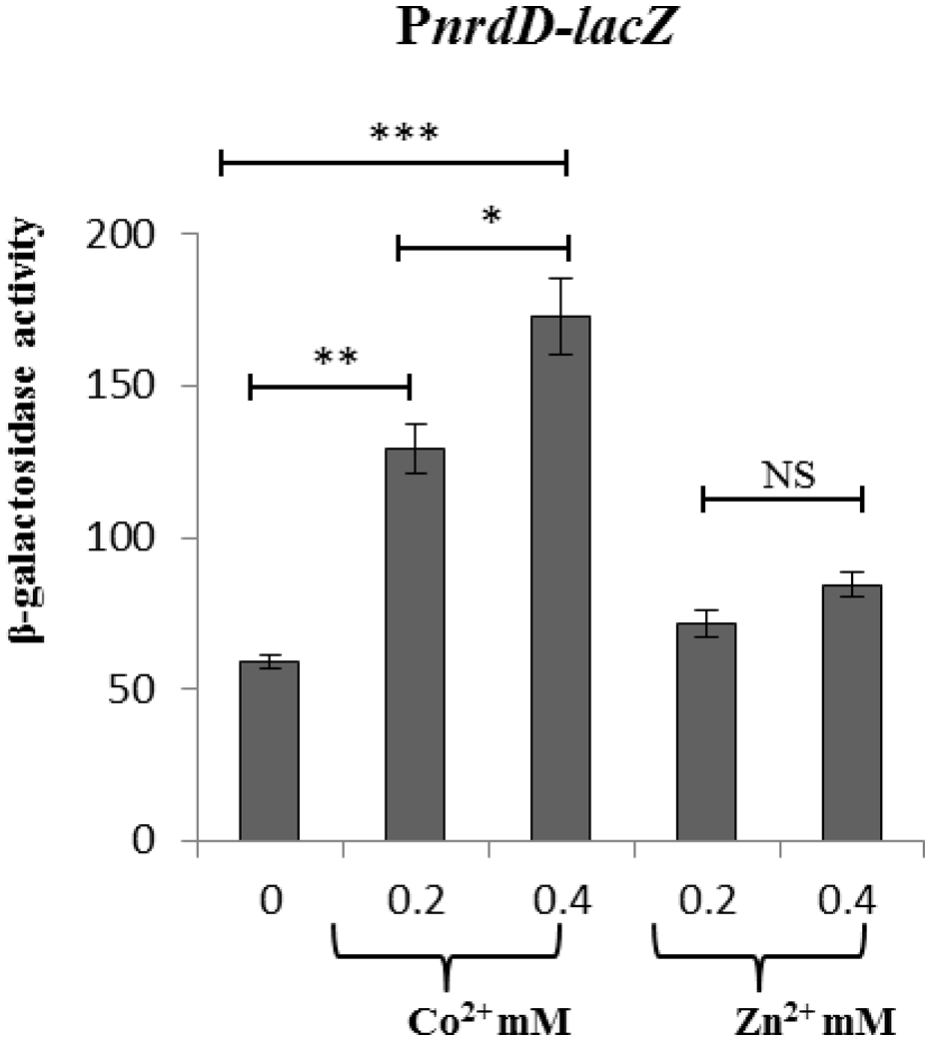
**Expression level (in Miller units) of P*nrdD-lacZ* in D39 WT in CDM supplemented with different concentrations of Co^2+^ and Zn^2+^.** SD of three independent replications is indicated with error bars. Statistical significance of the differences in the expression levels was determined by one-way ANOVA (NS, not significant, ^∗^*P* < 0.05, ^∗∗^*P* < 0.001, and ^∗∗∗^*P* < 0.0001).

Ribonucleotide reductases (*nrd*) usually catalyze the formation of deoxyribonucleotides necessary for DNA synthesis and DNA repair in almost all living organisms (Jordan and Reichard, 1998). Proper regulation of the *nrd* operon seems important for the cell. In *E. coli* and *L. lactis*, NrdD forms a complex with NrdG for specific ribonucleotide reductase activity (Torrents et al., 2001). In *E. coli*, the regulation of the *nrd* operon is mediated by two transcription factors, FNR and ArcA (Boston and Atlung, 2003). The *nrd* operon is highly upregulated in our transcriptome data under Co^2+^ stress and in previous studies also under Zn^2+^ and Cu^2+^ stress thus inhibiting the aerobic dNTP biosynthetic pathway (Kloosterman et al., 2008; Johnson et al., 2015). To validate our transcriptome data, we investigated the transcriptional response of P*nrdD-lacZ* to different concentrations of Co^2+^ and Zn^2+^ in CDM. Measurement of β-galactosidase activity showed that expression of P*nrdD-lacZ* is increased with increasing concentrations of Co^2+^ and Zn^2+^ (**Figure 5**). Expression of P*nrdD-lacZ* was twofold to threefold lower in the presence of Zn^2+^ as compared to Co^2+^. For future studies, it will be interesting to investigate what regulatory pathways govern the metal-dependent regulation of the *nrd* operon.

Previously, it has been shown that expression of P*czcD* is increased by the presence of Co^2+^ in the undefined rich growth medium GM17, which was dependent on the metal-dependent activator SczA (Kloosterman et al., 2007). In addition, CzcD was found to contribute to resistance of cells to a high concentration of Co^2+^. We confirmed these results in the CDM medium that was used throughout this study (Supplementary Figure S6).

## Discussion

In this study, we have explored Co^2+^-dependent gene expression in the human pathogen *S. pneumoniae* by whole transcriptome analysis. Several genes and operons with diverse functions were differentially expressed, including the regulon of the Mn^2+^-dependent transcriptional regulator PsaR, which is relevant for virulence, the Zn^2+^-resistance gene *czcD*, the putative Co^2+^-uptake operon *cbi*, and the *nrd* operon encoding genes involved in deoxyribonucleotides synthesis. The microarray results were confirmed by *lacZ*-reporter studies. Moreover, ICP-MS analysis showed that the transcriptional changes observed under Co^2+^ stress correlate with a high cell-associated Co^2+^ concentration, but not with changes in the concentration of other metal ions. Based on our *in vitro* studies of the interaction of PsaR with its target promoters, we propose that Co^2+^ leads to derepression of the regulon of the Mn^2+^-dependent transcriptional regulator PsaR by stimulating binding to its target promoters in a way that does not lead to repression as occurs with Mn^2+^.

Here, we present evidence that expression of the *cbi* operon, which is likely involved in the transport of Co^2+^, depends on the availability of Co^2+^. We could not obtain a deletion mutant of the *cbi* genes to study their effect on Co^2+^ homeostasis, probably because of polar effects on the essential downstream genes *mreCD*. However, the fact that the *cbi* genes are upregulated during Co^2+^ stress might indicate that their function is Co^2+^ export. This would mean that *S. pneumoniae* protects itself against Co^2+^ stress both *via* CzcD and the Cbi system. So far, a mechanism governing regulation of the *cbi* operon or similar genes in other organisms has not been studied. It is therefore interesting to search for a transcriptional regulator that mediates the Co^2+^-responsive expression of the *cbi* operon in *S. pneumoniae*. Of note, a putative transcriptional regulator is encoded in the *cbi* operon, and future experiments could be important to investigate whether this regulator is responsible for the Co^2+^-sensitive expression of the *cbi* operon. It is remarkable as well that two important cell division genes, namely *mreC* and *mreD*, are in the same operon with the *cbi* genes and that its expression is also Co^2+^ responsive. Although BLAST searches did not reveal linkage of *mreCD* to the *cbi* genes in other bacterial species, it would be interesting to investigate whether there is any biological reason for the presence of these genes in the same operon in *S. pneumoniae*.

Previously, it was shown that only Mn^2+^ and Zn^2+^ are responsible for the PsaR-mediated expression of the virulence genes *psaBCA*, *pcpA*, and *prtA* (PsaR regulon) and no role of Co^2+^ in the regulation of these genes was observed (Kloosterman et al., 2008). The previous study was done in the complex nutrient broth GM17 as a growth medium where Co^2+^ somehow was unable to stimulate expression of the PsaR regulon. GM17 may contain Co^2+^-chelating compounds that obscure the Co^2+^-dependent derepressive effect on the PsaR regulon. In the present work, we have demonstrated the role of Co^2+^ in the regulation of the PsaR regulon by using, instead of the complex GM17 medium, a CDM.

It has been shown before that competition between Mn^2+^ and Zn^2+^ results in Mn^2+^ deficiency in the cell possibly due to the involvement of the Mn^2+^-uptake transporter protein PsaA (Jacobsen et al., 2011). Additionally, it has been reported that PsaA has an ability to bind with other transition metal ions (Couñago et al., 2014), and Zn^2+^ and Cd^2+^ have the ability to inhibit Mn^2+^-uptake *via* PsaA (Eijkelkamp et al., 2014; Begg et al., 2015). Here, we performed ICP-MS analysis to check the intracellular concentration of Mn^2+^ in the presence of Co^2+^. Our ICP-MS analysis results showed that the intracellular concentration of Mn^2+^ is not affected by the addition of Co^2+^. This suggests that the expression of *pcpA*, *psaBCA*, and *prtA* is not derepressed by Co^2+^ due to Mn^2+^ starvation (Jacobsen et al., 2011), but instead might be a direct effect of an elevated intracellular Co^2+^ concentration.

Metal binding transcription factors can often bind to different metal ions and can therefore be at the cross-road of interplay between metal ions. For example, in *B. subtilis* CzrA is activated in the presence of Zn^2+^, while repressed in the presence of Cu^2+^ (Harvie et al., 2006). Similarly, the Cu^2+^-responsive regulator CopY regulates the expression of the *cop* operon in a Cu^2+^ and Zn^2+^-dependent way (Shafeeq et al., 2011b). Previously, the expression of P*pcpA-lacZ*, P*psaB-lacZ*, and P*prtA-lacZ* has been shown to depend on the balance between the Mn^2+^ and Zn^+2^ concentrations, where Zn^2+^ derepresses the expression of these gene/operons and Mn^2+^ nullifies this expression *via* PsaR in GM17 medium (Kloosterman et al., 2008). Here, working with CDM, we have shown that the proper regulation of PsaR regulon is not only dependent on the balance between Mn^2+^ and Zn^2+^, but also on the balance between Mn^2+^ and Co^2+^. MntR, a DtxR family protein, represses the expression of a Mn^2+^-uptake system in *B. subtilis* (Que and Helmann, 2000). It has 15% sequence homology with PsaR in *S. pneumoniae* (Kloosterman et al., 2008). MntR also has the ability to bind with Cd^2+^, Zn^2+^, Ni^2+^, Cu^2+^, and Co^2+^ (Lieser et al., 2003; Golynskiy et al., 2005, 2007). Recent structural studies showed that Co^2+^ binding to MntR prevents the binding of Mn^2+^ (McGuire et al., 2013). The metal ion binding residues of MntR (D8, E99, E102, and H103) are also present in PsaR (D7, E99, E102, and H103), (Kloosterman et al., 2008; Lisher et al., 2013). Because the metal binding residues of MntR are conserved in PsaR, it is likely that Co^2+^ can also inhibit binding of Mn^2+^ to PsaR in an analogous way. Since Co^2+^, like Mn^2+^, stimulates binding of PsaR to its target promoters, we propose that this PsaR-Co^2+^ interaction with the promoter is ineffective in terms of repression of expression. Possibly, in the presence of Co^2+^, PsaR binds to the promoters of *pcpA*, *prtA*, and *psaBCA*, in such a way that it leads to derepression of the PsaR regulon. Whether the effects between Co^2+^ and Mn^2+^ as described in our work is, in conjunction with previously described interaction between Zn^2+^ and Mn^2+^ and Cd^2+^ and Mn^2+^ (Jacobsen et al., 2011; Begg et al., 2015), relevant for the *in vivo* situation, i.e., infection of the human body, remains a topic for future investigation.

## Conflict of Interest Statement

The authors declare that the research was conducted in the absence of any commercial or financial relationships that could be construed as a potential conflict of interest.
