# Supplementary material for: Co2+-dependent gene expression in Streptococcus pneumoniae: opposite effect of Mn2+ and Co2+ on the expression of the virulence genes psaBCA, pcpA, and prtA
Source: Front Microbiol. 2015 Jul 24;6:748. doi: 10.3389/fmicb.2015.00748 (PMC4513243; doi:10.3389/fmicb.2015.00748)
Supplement: Supplementary file 1 [file Data_Sheet_1.DOCX]

**Supplementary Data**


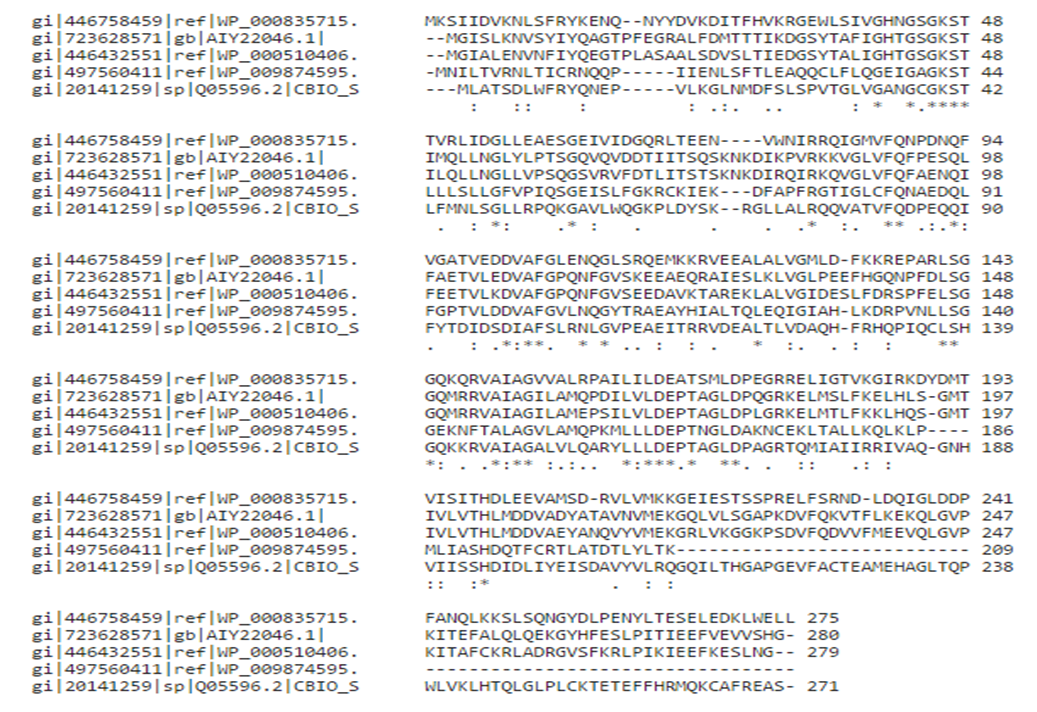


**Figure S1:** Sequence alignment of putative cobalt transport protein in *S. pneumoniae* D39 CbiO1 (WP_000835715.1) and CbiO2 (WP_000510406.1) with cobalt transport proteins of *Actinobacillus pleuropneumoniae* (WP_009874595.1)*,* *Streptococcus salivarius* (AIY22046.1) and *Salmonella typhimurium* (Q05596.2).


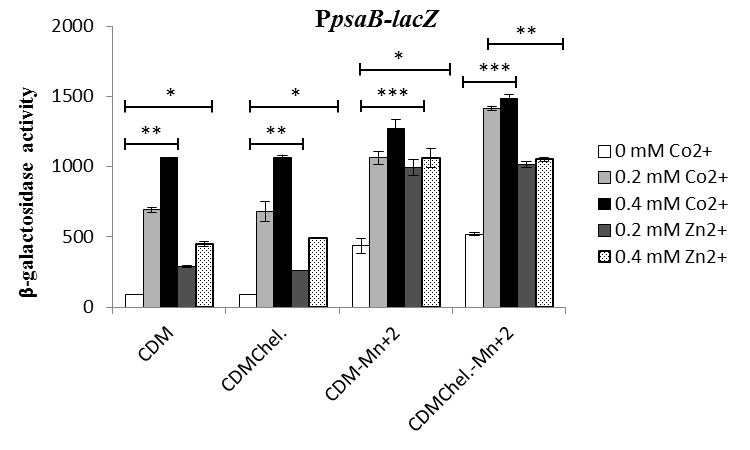


**A**


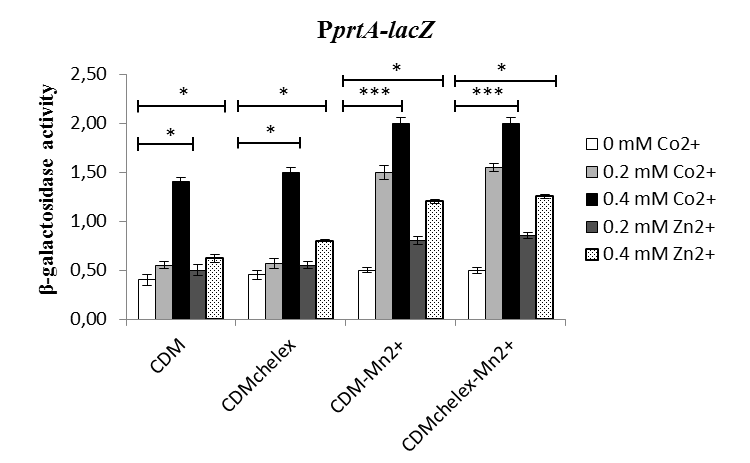


**B**

**Figure S2:** (**A)** Expression level (in Miller units) of P*psaB-lacZ* in D39 wild-type in CDM, CDM-Mn^2+^, CDMchelex and CDMchelex-Mn^2+^ supplemented with different concentrations of Co^2+^ and Zn^2+^. (**B)** Expression level (in Miller units) of a P*prtA-lacZ* in D39 wild-type in CDM, CDM-Mn^2+^, CDMchelex and CDMchelex-Mn^2+^ supplemented with different concentrations of Co^2+^ and Zn^2+^. Standard deviation of three independent replications is indicated with error bars. Statistical significance of the differences in the expression levels was determined by one-way ANOVA (NS, not significant, *P<0.05, **P<0.001 and ***P<0.0001).


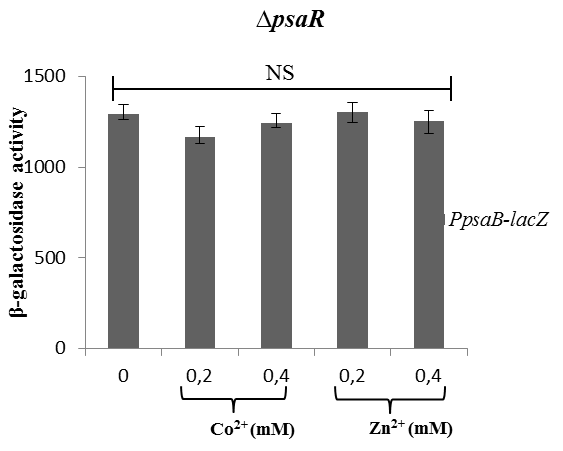


**A**


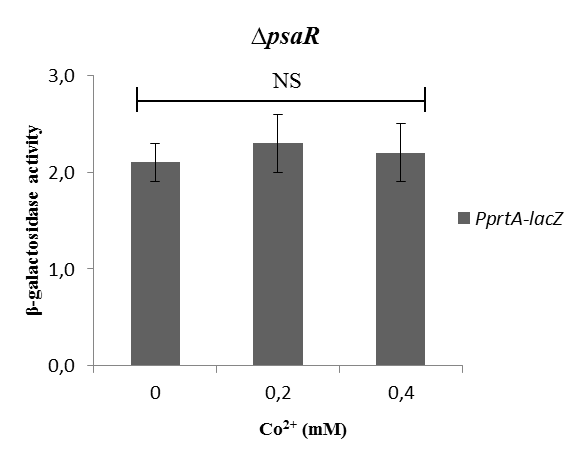


**B**

**Figure S3:** Expression level (in Miller units) of P*psaB-lacZ* (**A)** and P*prtA-lacZ* (**B)** in Δ*psaR* in CDM. *prtA-lacZ* was constructed in pORI13 integrated at the native chromosomal location. This might explain the lower Miller Units as compared to *lacZ*-fusions with P*pcpA* and P*psaB* (Fig. S2 (A)). Standard deviation of three independent replications is indicated with error bars. Statistical significance of the differences in the expression levels was determined by one-way ANOVA (NS, not significant).


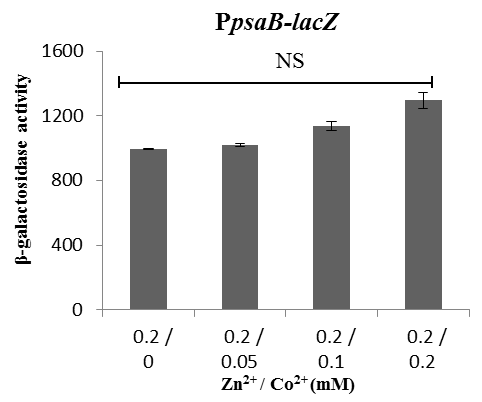


**A**


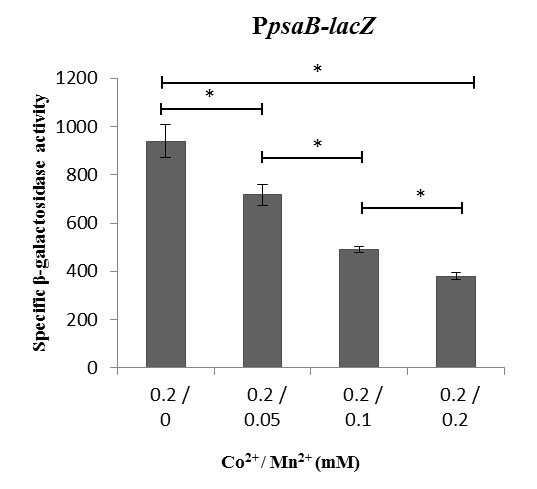


**B**


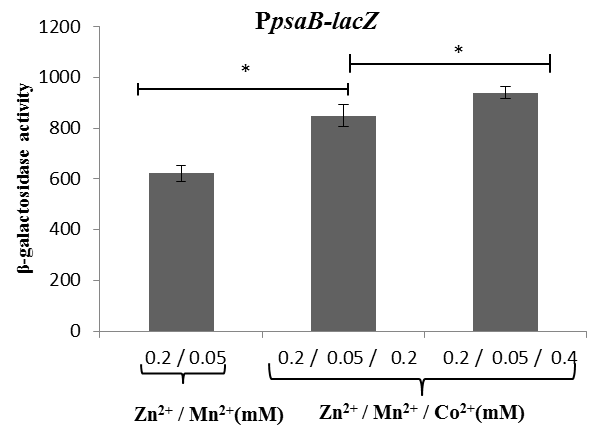


**C**

**Figure S4:** Expression level (in Miller units) of a P*psaB-lacZ* in D39 wild-type in CDM (without Mn^2+^ addition) supplemented with different concentrations of Co^2+^ and Zn^2+^ **(A)**, with different concentrations of Co^2+^ and Mn^2+^ **(B)**, and with different concentrations of Co^2+^, Mn^2+^and Zn^2+^ **(C)**. Standard deviation of three independent replications is indicated with error bars. Statistical significance of the differences in the expression levels was determined by one-way ANOVA (NS, not significant and *P<0.05).


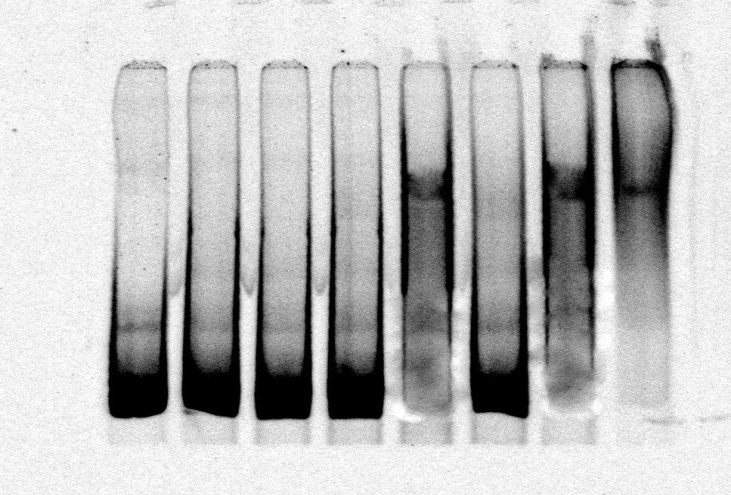

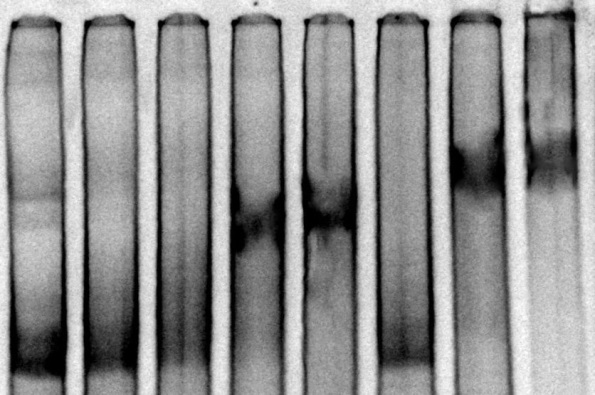


**PsaR-Strep**

**Co^2+^**

**Mn^2+^**

**1**

**2**

**3**

**4**

**5**

**6**

**7**

**8**

**x**

**A**

**B**

**Figure S5:** *In vitro* interaction of PsaR-Strep with the promoter regions of *prtA* (**A**) and *psaB* (**B**). PsaR-Strep was added at a concentration of 30nM as indicated with horizontal bar above the lanes, while lane 1 is without protein. Arrows indicate the position of shifted prove and asterisks indicate the position of free probe. Mn^2+^ was added in concentrations of 0.05 mM, 0.1 mM and 0.2 mM as indicated by the triangular bar above lanes 3, 4 and 5. Co^2+^ was added in concentrations of 0.05 mM, 0.2 mM and 0.4 mM as indicated by the triangular bar above lanes 6, 7 and 8 respectively.

**A**


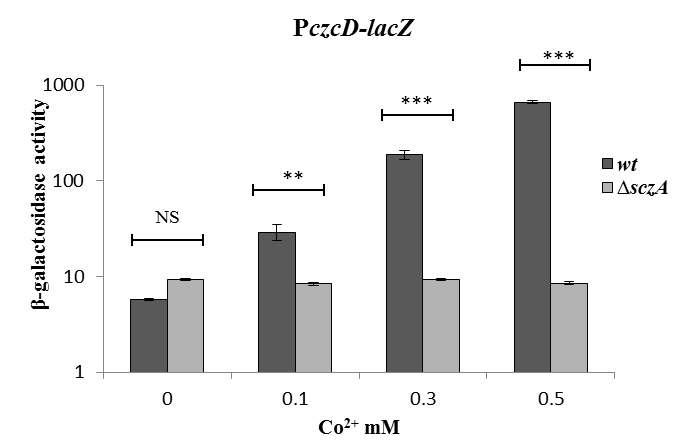


**B**

**Figure S6: (A)** Expression level (in Miller units) of a P*czcD-lacZ* in D39 wild-type and Δ*sczA* in CDM supplemented with different concentrations of Co^2+^. The standard deviation of three independent replications is indicated with error bars. Statistical significance of the differences in the expression levels was determined by one-way ANOVA (NS, not significant, *P<0.05, **P<0.001 and ***P<0.0001). (**B)** *S. pneumoniae* wild-type D39 and Δ*czcD* grown in CDM with 0mM and 0.5mM Co^2+^.

**Table S1:** The relative expression of *prtA*, *psaB*, *psaC*, *psaA* and *pcpA* genes. The expression of *prtA*, *psaB*, *psaC*, *psaA* and *pcpA* genes was normalized with the housekeeping gene *gyrA*. Standard deviation of three independent replications is given in parentheses. The 2 log fold increase is relative to the expression in the D39 grown in 0.4 mM Co^2+^ to that with 0 mM Co^2+^.

| **Gene tag** | **Gene function** | **Fold Raito** |  |
| --- | --- | --- | --- |
| *spd_0558* | Cell wall-associated serine protease PrtA | 5.12 (1.23) | |
| *spd_1461* | Manganese ABC transporter, ATP-binding protein | 5.70 (0.63) | |
| *spd_1462* | Manganese ABC transporter, permease protein, putative | 6.49 (1.74) | |
| *spd_1463* | Manganese ABC transporter, ATP-binding protein | 5.68 (1.43) | |
| *spd_1965* | Choline binding protein PcpA | 7.59 (1.73) | |

**Table S2:** Metal ion concentrations (µg l^-1^) in CDM and chelex treated CDM with and without the addition of Metal mixture.

| Metal ions | Complete CDM | Complete CDM Chelex treated | CDM without metal mix | CDM without metal mix Chelex treated |
| --- | --- | --- | --- | --- |
| Mn^2+^ | 531.2 | 528.5 | 5.1 | 2.3 |
| Zn^2+^ | 883 | 520 | 4.2 | 3.7 |
| Co^2+^ | 479.3 | 293.6 | 11.9 | 4.6 |
| Fe^2+^ | 30 | 11.5 | 30.7 | 12.7 |
| Ni^2+^ | 4.6 | 2.4 | 1.9 | 0.3 |

**Materials and Methods**

**RNA isolation and cDNA synthesis for qRT-PCR**

**Quantitative real time (qRT)-PCR experiments**

For qRT-PCR, S. pneumoniae D39 wild-type was grown in CDM with 0 mM Co^2+^ and 0.5 mM Co^2+^ till mid-exponential phase. RNA isolation was performed as described before (Shafeeq et al., 2011). Additionally, RNA samples were re-treated with DNase I (RNase-free) (Thermo Fisher Scientific, St. Leon-Rot, Germany) for 60 minutes at 37 °C in DNase I buffer (10 mM of Tris HCl (pH 7.5), 2.5 mM of MgCl_2_, 0.1 mM of CaCl_2_). cDNA synthesis was performed on 4 µg RNA using superscript III reverse transcriptase (Invitrogen) and random nonamers at 42 °C for 55 min. An iQ5 Real-Time PCR System (BioRad, Hercules, CA) was used to Quantify cDNA (1µl) using 1 x CYBR Green PCR master mix (Applied biosystems, Foster City, USA) and 3 pmol of each primer (Table2). The following cycling parameters were used for the reaction: 1 cycle of 10 min 95 °C followed by 40 cycles of 30 s 95 °C, 1min 55 °C, and 30 s 72 °C. PCR reactions were performed in triplicate for each cDNA sample. The transcription level of the target genes was normalized to gyrA transcription using the relative expression software tool (Pfaffl, 2001).

**Table 3.** List of primers used for qRT-PCR.

| **Name** | **Nucleotide Sequence (5’🡪3’)** |
| --- | --- |
| prtA-F | GCAGCCTATGCCCCTAATG |
| prtA-R | GTTTTAGTGTCTATTACAGG |
| pcpA-F | CCAATCCTAGCAGATACTCC |
| pcpA-R | GTAGGAATCGTGAATGG |
| psaB-F | CCTCAGTGTCTCCTACAAAG |
| psaB-R | GGCAATTCGGTGTAAGG |
| psaC-F | CCATTTCCTACAAAATGCCTT |
| psaC-R | TCCAAAGACAATGGCTCC |
| psaA-F | CTCGTTCTCTTTCTTTCTG |
| psaA-R | CTTAACGTCTTCAGGAA |
| gyrA-F | CGAGGCACGTATGAGCAAGA |
| gyrA-R | GACCAAGGGTTCCCGTTCAT |

**References**

Pfaffl, M. W. (2001). A new mathematical model for relative quantification in real-time RT-PCR. *Nucleic Acids Res.* 29, e45.

Shafeeq, S., Yesilkaya, H., Kloosterman, T. G., Narayanan, G., Wandel, M., Andrew, P. W., Kuipers, O. P., and Morrissey, J. A. (2011). The cop operon is required for copper homeostasis and contributes to virulence in Streptococcus pneumoniae. *Mol. Microbiol.* 81, 1255–1270. doi:10.1111/j.1365-2958.2011.07758.x.
